# Effectiveness and safety of Korean medicine treatment based on the clinical practice guidelines in patients with acute peripheral facial palsy: A protocol for a multicenter, prospective, observational study

**DOI:** 10.1097/MD.0000000000029864

**Published:** 2022-07-08

**Authors:** Hyewon Lim, Yoonji Lee, Suji Lee, Yong-Suk Kim

**Affiliations:** a Department of Clinical Korean Medicine, Graduate School, Kyung Hee University, Seoul, Republic of Korea; b Department of Acupuncture and Moxibustion, Kyung Hee University Korean Medicine Hospital, Seoul, Republic of Korea; c Department of Acupuncture and Moxibustion, College of Korean Medicine, Kyung Hee University, Seoul, Republic of Korea.

**Keywords:** acute peripheral facial palsy, Korean medicine treatment, Korean medicine clinical practice guideline

## Abstract

**Introduction:**

Peripheral facial palsy (PFP) results in weakness or paralysis of the affected side of the face. In Korea, there is a high demand for Korean medicine treatment for PFP. The clinical practice guidelines (CPGs) of Korean medicine for facial palsy were developed; however, there remains insufficient evidence to support the effectiveness and safety of Korean medicine treatment. Thus, this study aimed to evaluate the effectiveness and safety of Korean medicine treatment based on the CPGs in patients with acute PFP.

**Methods:**

This is a multicenter, prospective, observational study. The participants will be recruited from one Korean medicine hospital and eight Korean medicine clinics. The participants will receive Korean medicine treatments based on the CPGs, fill in survey questionnaires, and undergo electrophysiologic testing. The changes in House-Brackmann (H-B) grade, movement of the lip and eye, symptoms related to or accompanied by facial palsy, Facial Disability Index, EuroQol 5-dimension 5-level (EQ-5D-5L), and EuroQol Visual Analogue Scale (EQ-VAS), and the results of electromyography (EMG), electroneurography (ENoG), and Blink Reflex test will be analyzed. For the safety analysis, adverse events will be recorded, and for the feasibility analysis, the results of the Was It Worth It questionnaire will be assessed.

**Conclusion:**

We expect to draw real-world clinical data on the effectiveness and safety of Korean medicine treatment based on the CPGs in patients with acute PFP from this study. It would be the basis for complementing and improving the CPGs and provide the basis of clinical and policy decision-making.

**Trial registration:**

This study was approved by the Institutional Review Board of Kyung Hee University Korean Medicine Hospital (2021-06-005-001), and registered with the Korean Clinical Trial Registry (CRIS), Republic of Korea (KCT0006562).

## 1. Introduction

The facial nerve controls most of the muscles in the face and parts of the ear, and it also provides innervation to the lacrimal and salivary glands, as well as taste to the anterior two-thirds of the tongue.^[1]^ Peripheral facial palsy (PFP) generally refers to facial muscle weakness, which may lead to permanent damage to the facial nerve. Besides incomplete facial movement, patients may complain of other symptoms, such as postauricular pain, increase or decrease in tear, taste disorder, and hyperacusis. These symptoms and signs typically peak in the first week and then gradually resolve. Oral corticosteroids have traditionally been prescribed to reduce facial nerve inflammation, and because of the possible role of Herpes simplex virus 1 in the etiology, antiviral drugs can be given.^[2]^

According to the 2019 National Health Insurance Statistical Yearbook in Korea, a total of 111,089 patients visited Korean medicine hospitals and clinics due to facial nerve disorder, ranking 26th among all diseases.^[3]^ Although there is no recommendation regarding the effect of acupuncture in clinical practice guidelines (CPGs) of the American Academy of Otolaryngology-Head and Neck Surgery (AAO-HNSF) for Bell’s palsy^[4]^ and Cochrane review,^[5]^ many patients with PFP in Korea still visit Korean medicine hospitals and clinics for acupuncture and other Korean medicine treatments.

Because there is a high demand for Korean medicine treatment for PFP, the need for the CPGs of Korean medicine for PFP has emerged, which could be the standard for creating the basis of the effectiveness and safety of Korean medicine treatment in the real-world setting. Public clinical trials have been conducted to support the development of CPGs and produce real-world evidence in the healthcare field; however, public clinical trials conducted at the primary medical institution level are not enough. Additionally, in 2019, the CPGs of Korean medicine for facial palsy (FP)^[6]^ were developed, but there remains insufficient evidence to support the effectiveness and safety of Korean medicine treatment. Therefore, it is necessary to continuously supplement and improve the CPGs and strengthen the coverage of Korean medicine treatment within the current medical system.

In this study, patients with acute PFP will be recruited from 1 Korean medicine hospital and 8 Korean medicine clinics, and Korean medicine treatments will be provided based on the CPGs of Korean medicine for FP to identify the effectiveness and safety of Korean medicine treatment with real-world data.

## 2. Methods

### 2.1. Study design

This is a multicenter, prospective, observational study on Korean medicine treatment based on the CPGs in patients with acute PFP. PFP is diagnosed after excluding central facial palsy through symptoms and history taking. The participants will be recruited from 1 Korean medicine hospital and 8 Korean medicine clinics, from August 2021 to February 2022. To minimize selective bias, patients with facial palsy who visited the relevant medical institution are sequentially recruited and screened. The participants will receive Korean medicine treatments at each hospital or clinic, and fill in survey questionnaires from the baseline until six weeks later. The participants’ survey questionnaires include symptoms related to or accompanied by FP, Facial Disability Index (FDI), EuroQol 5-dimension 5-level (EQ-5D-5L), EuroQol Visual Analogue Scale (EQ-VAS), and Was It Worth It (WIWI) questionnaire. The participants will undergo electromyography (EMG), electroneurography (ENoG), and Blink Reflex test between visits 3 and 4, which is 2–3 weeks from the onset of PFP. The changes in House-Brackmann (H-B) grade and movement of the eyebrow and lip will be evaluated by investigators, and the treatments provided to the participants and adverse events will be recorded.

### 2.2. Inclusion criteria

The inclusion criteria are the following:

(1) Patients whose primary diagnosis is PFP (G51.0 Bell’s palsy; G51.8 Detailed facial neuropathy; G51.9 Facial nerve damage; and B022 Ramsay Hunt syndrome),
(2) Patients with FP that occurred within 7 days,
(3) Patients aged over 19 years.

### 2.3. Exclusion criteria

The exclusion criteria are the following:

(1) Patients participating in other clinical studies confirming the effectiveness of the intentional treatment,
(2) Patients with a previous history of FP,
(3) Patients with bilateral FP,
(4) Patients who received Korean medicine treatment through other Korean medicine clinics or Korean medicine hospitals,

(If patients receive Korean medicine treatment though other Korean medicine clinics or hospitals, the investigators are not able to know what kind of Korean medicine treatments are performed to the patients, and it is difficult to determine the effectiveness of the treatments.)

(5) Patients who are expected to have difficulty adapting to clinical research schedules,

(Patients who are not able to visit once a week were excluded.)

(6) Patients who are expected to have difficulty understanding and responding to research questionnaires due to illiteracy and mental or physical weaknesses,

(Patients who are not able to understand this study and read and response the questionnaires were excluded.)

(7) Patients who are determined by the researchers to be unable to participate in the study.

### 2.4. Sample size

Based on similar studies, the clinical experiences of the investigators, and the preliminary research results,^[7,8]^ the sample size was determined. In this study, the therapeutic effect is defined as H-B grade 2 or lower. Assuming that the ratio of the patients with H-B grade 2 or lower after the treatments for 6 weeks is 80% (±10%, *P* = .05), considering the power of 80% and dropout rate of 15%, a total of 73 participants will be required. There were 2708 patients with PFP from 2006 to 2015 in Korea, and the average incidence rate over 10 years was 25.9 cases per 100,000 people.^[9]^ Therefore, the calculated 73 study subjects can be sufficiently recruited for the study period of about 6 months.

### 2.5. Ethics

This study will be conducted in accordance with the Declaration of Helsinki and the Guidelines for Good Clinical Practice. Before obtaining consent, all patients will be provided with oral and written study information. This study was approved by the Institutional Review Board of Kyung Hee University Korean Medicine Hospital (KOMCIRB 2021-06-005-001) and registered with the Korean Clinical Trial Registry (CRIS), Republic of Korea (KCT0006562).

### 2.6. Consent and registration

The participants will be recruited from 1 Korean medicine hospital and 8 Korean medicine clinics. They will be provided with study information. After providing consent to the enrollment, eligibility criteria will be evaluated, and the participants will be assigned an identification number and registered as study participants. Registration will be closed when the planned number of participants has been reached.

### 2.7. Treatment

The participants will receive Korean medicine treatments based on the CPGs but will not receive any intentional treatments. The Korean medicine treatments may include acupuncture, electroacupuncture, pharmacopuncture, moxibustion, herbal medicine, and cupping. The investigators will keep a record of the treatments provided to the participants.

According to the CPGs,^[6]^ acupoints such as ST4, ST6, EX-HN5, TE17, GB14, SI18, LI4, and GB20 are frequently used for acupuncture treatment for FP, and other acupoints can also be used according to the patient’s symptoms and judgment of Korean medicine doctors. In addition, electrical stimulation can be applied to the acupoints. In case of herbal medicine treatment, Korean medicine doctors determine the prescription according to the condition and characteristics of each patient. Acupoints such as TE17 and GB12 can be used for wet cupping. Pharmacopuncture such as Bee venom and placenta, and moxibustion treatment can be applied on face and behind the ear.

### 2.8. Data collection

Data will be obtained by investigator assessment and participant survey at baseline and at specified follow-up times (Table 1).

**Table 1:** Schedule of observations

		Observation Period						
Visit	Screening	1	2	3	4	5	6	7
**Week**	**0-1**	**0-1**	**1**	**2**	**3**	**4**	**5**	**6**
**Informed consent**	**●**							
**Demographics**	**●**							
**Vital sign and body measurement**	**●**							
**Medical history of facial palsy**	**●**							
**Medical history of underlying disease and medication**	**●**							
**Inclusion/Exclusion criteria**	**●**							
**House-Brackmann grading system**		**●**	**●**	**●**	**●**	**●**	**●**	**●**
**Movement of eyebrow and lip**		**●**	**●**	**●**	**●**	**●**	**●**	**●**
**Symptoms related to facial palsy**		**●**	**●**	**●**	**●**	**●**	**●**	**●**
**Symptoms accompanied by facial palsy**		**●**	**●**	**●**	**●**	**●**	**●**	**●**
**FDI**		**●**						**●**
**QoL (EQ-5D-5L, EQ-VAS**)		**●**						**●**
**WIWI questionnaire**								**●**
**EMG, ENoG, Blink reflex**				**●**				
**Adverse events**		**●**	**●**	**●**	**●**	**●**	**●**	**●**

### 2.9. Investigator assessment

The investigators, who are Korean medicine doctors working in the hospital and clinics participating in this study, will assess the changes in H-B grade and movement of the eyebrow and lip. All measurements and assessments will be performed at baseline and once a week until 6 weeks later.

### 2.10. Participant survey

The participants will fill in survey questionnaires at baseline and once a week until 6 weeks later. The questionnaires include symptoms related to or accompanied by FP, FDI, EQ-5D-5L, EQ-VAS, and WIWI questionnaire. The participants will fill in the questionnaires about symptoms related to or accompanied by FP at every visit, FDI, EQ-5D-5L, and EQ-VAS at the first and last visits, and WIWI questionnaire only will be at the last visit.

### 2.11. Primary outcome measurement

The primary outcome is the changes in H-B grade from baseline until 6 weeks later. The H-B grading system is the most widely used and accepted scale for motor function assessment that helps document the degree of paralysis from grade I (normal facial function) to grade VI (total paralysis)^[10]^ (Table 2). The reliability of H-B grading system for FP has been proven, and it has been used as a simple and robust method of assessing facial function.^[11]^

**Table 2:** House-Brackmann grading system

Grade	Definition
I	Normal symmetrical function in all areas
II	Slight weakness noticeable only on close inspection
	Complete eye closure with minimal effort
	Slight asymmetry of smile with maximal effort
	Synkinesis barely noticeable, contracture, or spasm absent
III	Obvious weakness, but not disfiguring
	May not be able to lift eyebrow
	Complete eye closure and strong but asymmetrical mouth movement with maximal effort
	Obvious, but not disfiguring synkinesis, mass movement or spasm
IV	Obvious disfiguring weakness
	Inability to lift brow
	Incomplete eye closure and asymmetry of mouth with maximal effort
	Severe synkinesis, mass movement, spasm
V	Motion barely perceptible
	Incomplete eye closure, slight movement corner mouth
	Synkinesis, contracture, and spasm usually absent
VI	No movement, loss of tone, no synkinesis, contracture, or spasm

### 2.12. Secondary outcome measurement

The secondary outcomes include the changes in the movement of the eyebrow and lip, symptoms related to or accompanied by FP, FDI, EQ-5D-5L, EQ-VAS, and the results of EMG, ENoG and Blink Reflex test, as well as the safety and feasibility analyses.

The movement of the eyebrow and lip will be measured at the top of the eyebrow and the corner of the mouth from each side, and the lengths will be compared.

Symptoms related to FP, including fatigue or overwork, poor sleep, stress, indigestion, loss of appetite, and aversion to cold or wind, will be examined on a checklist. Additionally, symptoms accompanied by FP, including postauricular pain, increase in tear, dry eye, ear fullness, hyperacusis, taste disorder, paresthesia of the tongue, and vesicle, will be evaluated using a 3-point Likert scale.

FDI is a disease-specific, self-report instrument for the assessment of disabilities of patients with facial nerve disorders, including physical function and social/well-being function. It has been proven that FDI subscales produce reliable measurements, with construct validity for measuring patient-focused disability of individuals with disorders of the facial motor system.^[12]^

The EQ-5D is a generic instrument for describing and valuing health based on a descriptive system, which defines health in terms of 5 dimensions: mobility, self-care, usual activities, pain/discomfort, and anxiety/depression.^[13]^ The EQ-5D-5L includes 5 levels of severity in each of the existing 5 EQ-5D dimensions,^[14]^ which provides precise and sensitive measurements of health status.^[15]^ The EQ-VAS is a vertical, calibrated line, bounded at 0 “worst imaginable health state” and 100 “best imaginable health state,”^[16]^ which exhibits sufficient construct validity.^[17]^

The EMG, ENoG, and Blink Reflex test are useful indicators for predicting recovery and defective healing with high prognostic value. It is known that the timing of electrophysiologic tests is important in order to predict the prognosis accurately.^[18]^ It takes about 72 hours for Wallerian degeneration to propagate from intratemporal part, which is the damaged part, to stylomastoid foramen, which is the distal part electrically stimulated during electrophysiologic tests.^[19]^ Reliable prognostic information is obtainable after 10–14 days from the onset of PFP.^[20]^ Electrophysiologic tests will be conducted 2–3 weeks from the onset at the Department of Physical Medicine of Rehabilitation in Kyung Hee University Hospital.

The safety analysis will be conducted by estimating the number of participants with and without serious adverse events (SAEs) during the study. If adverse event occurs, the investigators will record it in detail in the case report form. If participants complain of side effects or abnormal reactions, or if the progress of the study threatens the safety of the participants, the investigators may decide to suspend or prematurely terminate the study for those participants, and report these to Institutional Review Board.

The WIWI questionnaire is a 5-item tool used to evaluate the feasibility of a clinical study and explores the patient’s experience of an intervention.^[21]^ With reference to previous research,^[21]^ it is meaningful to conduct the questionnaire after the participants receive Korean medicine treatment. The participants will fill in the WIWI questionnaire at the end of the study, and the results will be analyzed to assess the feasibility of Korean medicine treatments.

### 2.13. Statistical analysis

For the primary outcome measure, including the H-B grading system, the proportion, and ratio of patients with H-B grade 2 or lower after the treatments will be analyzed.^[22,23]^

For the secondary outcome measures, including the movement of the eyebrow and lip, symptoms related to or accompanied by FP, FDI, EQ-5D-5L, EQ-VAS, WIWI questionnaire, and the results of EMG, ENoG, and Blink Reflex test, data will be analyzed using the mixed-effect model repeated measure, with visit time as a fixed factor and subject as a random factor.

Prognosis will be estimated using the results of EMG, ENoG and Blink Reflex test, and the correlation between the prognosis and the changes in H-B grade, movement of the eye and lip, symptoms related to or accompanied by FP, or the types of Korean medicine treatments provided will be analyzed using logistic regression analysis, considering age and sex as a potential confounders.

Since various Korean medicine treatments will be used, all treatments provided to the subjects will be recorded, and adverse events will be evaluated at every visit. For the safety analysis, the number of participants with and without SAEs will be estimated. The degree of symptoms and relevance to treatments will be analyzed, and SAE details will be described.

## 3. Discussion and Conclusion

PFP causes significant changes in facial function and appearance. It can lead to difficulties with facial expression, eating, drinking, hearing, or speaking and can have a negative impact on psychological well-being, social function, and quality of life (QoL).^[24,25]^

In Korea, the demand for Korean medicine treatment for PFP is high, and many patients with PFP visit Korean medicine hospitals and clinics to be treated.^[3]^ Public clinical trials have been conducted, which systematically collect and analyze patient data to evaluate the effectiveness and safety of the medical technologies, and provide the basis for clinical and policy decision-making. The results are applied to healthcare policies, along with strengthening the coverage and improving the medical quality.

The CPGs of Korean medicine for FP were developed and have been revised and supplemented. However, in a recent study, it has been reported that evidence supporting the efficacy and safety of acupuncture is lacking due to the poor quality and heterogeneity of relevant studies,^[26]^ and further researches are required to provide an appropriate level of evidence. Thus, more studies and data are needed to evaluate the effectiveness and safety of Korean medicine treatment, complement and improve the CPGs, and finally increase the coverage of Korean medicine treatment.

According to previous study,^[27]^ as there are limitations of conventional medicine treatment in FP, there are increased interest in Korean medicine treatment. Acupuncture, electroacupuncture, pharmacopuncture, and herbal medicine are mainly used to treat FP. This study aims to evaluate the effectiveness and safety of Korean medicine treatment for patients with acute PFP, when applied to the real-world clinical environment. We expect that the study findings might provide real-world clinical data to be used as the basis of clinical and policy decision-making.

## Author contributions

Conceptualization: Hyewon Lim and Yong-Suk Kim

Funding acquisition: Yong-Suk Kim

Investigation: Yoonji Lee and Hyewon Lim

Methodology and project administration: Hyewon Lim, Yoonji Lee, Suji Lee, and Yong-Suk Kim

Supervision: Yong-Suk Kim

Writing–original draft: Hyewon Lim

Writing–review and editing: Suji Lee and Yong-Suk Kim
